# Anthocyanin accumulation differences in European pears caused by *Phytochrome-interacting factor 3 (PcPIF3)* promoter mutations under UV-B

**DOI:** 10.1016/j.jare.2025.05.010

**Published:** 2025-05-13

**Authors:** Haowei Cao, Yingying Qu, Lei Guo, Mengjia Wu, Guorong Zhang, Ying Tang, Hongjuan Zhang, Rui Zhai, Chengquan Yang, Lingfei Xu, Zhigang Wang

**Affiliations:** a State Key Laboratory of Crop Stress Biology for Arid Areas, Northwest A&F University, Taicheng Road No. 3, Yangling, Shaanxi Province, China; b Hangzhou Gene Science-Medicals Co., Ltd., 5F, Kangfa S&T Park, Xiaoshan District, Hangzhou, Zhejiang Province, China

**Keywords:** *PcPIF3*, *PcWRKY11*, Anthocyanin, Pear, UV-B

## Abstract

The phenomenon of red fading in late-stage red-skinned pear fruit development significantly impacts its value. This phenomenon also occurs in ’Starkrimson’, albeit to a lesser extent compared to ’Red Bartlett’ and other varieties. The reasons for the differences in anthocyanin accumulation during the late stages of fruit development in red-skinned European pears varieties are not yet understood. Here, we report a novel pattern of anthocyanin accumulation in response to UV-B in ’Starkrimson’ pears, which is associated with mutations in the *PcPIF3* promoter. Through transcriptome analysis of ’Red Bartlett’ and ’Starkrimson’ pears, we identified the differentially expressed gene *PcPIF3*, which is capable of promoting anthocyanin biosynthesis through direct binding to the promoter region of *PcMYB10*. Under moderate UV-B conditions, the ’Starkrimson’ *PcPIF3* were significantly increased. By analyzing the *PcPIF3* promoter regions of different red-skinned pear varieties, we identified the differential binding element as W-BOX 1 and subsequently screened for the upstream positive regulator, PcWRKY11. Y1H and related experiments demonstrated that under UV-B exposure, PcWRKY11 binds specifically to the W-BOX 1 element in the ’Starkrimson’ *PcPIF3* promoter, thereby enhancing its transcription. These findings indicate that PcWRKY11, in response to UV-B, interacts with the W-BOX 1 element in the ’Starkrimson’ *PcPIF3* promoter, promoting anthocyanin biosynthesis and mitigating the fading of red coloration in ’Starkrimson’ pears. The difference in the *PcWRKY11-PcPIF3* module represents a novel mechanism for the variation in anthocyanin accumulation among different red-skinned pear varieties.

## Highlights

• PcPIF3 has been identified as playing a positive role in pear skin coloration.
• PcWRKY11 has been confirmed to respond to UV-B.
• Under UV-B exposure, PcWRKY11 regulates anthocyanin biosynthesis by activating the *PcPIF3* promoter.
• A *PcPIF3* promoter mutation creates an extra PcWRKY11 site, causing color differences in red-skinned European pears.

## Graphical abstract

**Figure:**
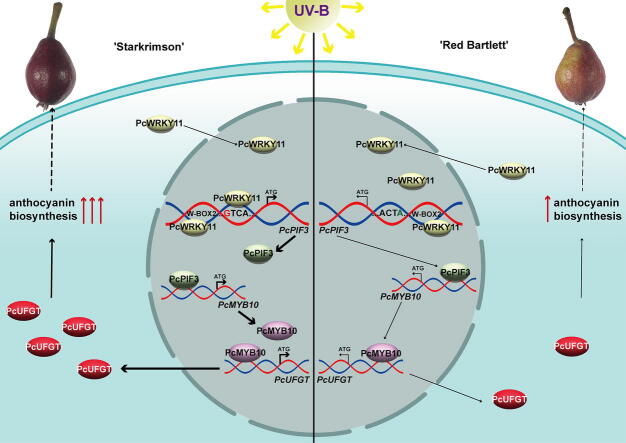

## Introduction

Red fruits are favoured by consumers due to their high nutritional value and vibrant colour [1]. Red-skinned European pears are the predominant red pear variety in the market, and their red fruit colour is attributed to anthocyanins [2]. Partial red-skinned European pear varieties exhibit fading of fruit colour during late-stage ripening, attracting attention to the anthocyanin accumulation patterns [3,4]. Anthocyanins, as secondary metabolites, are extensively distributed in various plant tissues, including flowers, fruits, leaves, and seeds [5]. They play a crucial role in enhancing colour diversity to attract pollinators and in increasing plant resilience to abiotic stresses such as UV-B, salt, and drought [6,7]. Anthocyanins also aid in resistance against pathogens and herbivores, thus facilitating plant adaptation to the environment [8]. As a natural water-soluble pigment, anthocyanins exhibit strong antioxidant properties, allowing them to neutralize free radicals in the body and slow down the aging process. Their significant health benefits make them valuable for human well-being [9,10].

The biosynthesis of anthocyanins is influenced by various environmental factors, with light being the most crucial determinant [11]. Extensive research has revealed a strong correlation between anthocyanin biosynthesis and the light signalling pathway [12]. The majority of studies suggest a positive correlation between light intensity and anthocyanin biosynthesis. However, some researchers also point out that excessive light intensity can inhibit anthocyanin accumulation [13]. The fruit colouring of red-skinned European pear is influenced by light intensity, but it does not show a direct proportionality with light density. Moreover, excessive light intensity can inhibit the colouring process [14]. Indeed, in addition to light intensity, UV-B radiation present in light can also impact the biosynthesis of anthocyanins [15]. Under appropriate UV-B radiation doses, the anthocyanin content in rice is negatively correlated with UV-B radiation dosage [16], but UV-B induces the accumulation of anthocyanins in maize [17]. Rose petals initiate anthocyanin biosynthesis upon exposure to UV-B light, while this process is suppressed in the absence of UV-B [18]. Further studies have demonstrated that UV-B-induced anthocyanin biosynthesis in radishes can occur even in darkness, indicating that UV-B can activate this process independently of light conditions [19]. As fruits mature, the inducibility of anthocyanin biosynthesis by UV-B diminishes in blueberries and apples [[20], [21], [22]]. Under higher light and UV-B irradiation, the synthesis of more flavonoids can be promoted in Asian pears [23].

Phytochrome-interacting factors (PIFs) [24] are a family of basic Helix-Loop-Helix (bHLH) proteins [25]. The bHLH proteins interact with MYB and WD40 proteins to form the MBW (MYB-bHLH-WD40) complex. [26]. This complex has been shown to regulate the transcription of several genes encoding structural proteins involved in anthocyanin biosynthesis [27,28]. The first one identified as a phytochrome interaction factor in the bHLH family is *PIF3*. The light response factor *PIF3* is a light response factor that can interact with phytochrome A (PhyA) and phytochrome B (PhyB) [29]. Overexpression of AtPIF3 in Arabidopsis leads to an increase in anthocyanin content. Further investigation revealed that AtPIF3 positively regulates anthocyanin biosynthesis through a mechanism that depends on AtHY5 [30,31]. On the other hand, MdPIF7 inhibits anthocyanin biosynthesis in apple [32]. PyPIF5 has also been reported to inhibit anthocyanin biosynthesis in pear by regulating the PySPL9 and PyMYB114/MYB10 transcription factors [33]. However, PpPIF8 can directly bind to the *proPpCHS*, thereby promoting anthocyanin accumulation in pear peel [34].

The WRKY gene family is one of the largest families of transcription factors (TFs). WRKY proteins are known to directly bind to the core W-box (TGACC(A/T)) of target genes to regulate their responses to stress [35]. WRKY71 has been reported to enhance anthocyanin biosynthesis in strawberries by upregulating the expression of structural genes involved in anthocyanin biosynthesis [36]. In apples, MdWRKY75 binds to the promoter region of MdMYB1, thereby regulating the expression of structural genes involved in anthocyanin biosynthesis [37]. Additionally, similar to MdWRKY11, MdWRKY75 can also promote anthocyanin biosynthesis by affecting the light-responsive factor MdHY5 [38]. In pears, WRKY transcription factors also play a role in promoting anthocyanin accumulation. PpWRKY44 directly binds to *PpMYB10* to promote anthocyanin biosynthesis [3], while PbWRKY75 not only indirectly regulates *MYB10b* but also directly regulates structural genes to promote anthocyanin biosynthesis [39].

Existing experiments have demonstrated that members of the PIFs family play complex roles in anthocyanin biosynthesis. Although AtPIF3 in Arabidopsis promotes anthocyanin biosynthesis, the function of PcPIF3 in pear remains unknown, and its regulatory network is unclear. Therefore, in this study, ‘Starkrimson’ and ‘Red Bartlett’ pears were utilized as materials to investigate the regulatory effect of *PcPIF3* on anthocyanin synthesis, as they exhibit distinct colouring patterns during the later stages of fruit development. Transient overexpression and silencing of *PcPIF3* on the pear peel indicated a positive role of *PcPIF3* in anthocyanin accumulation. In addition, we screened out upstream transcription factor PcWRKY11 of *PcPIF3* from the transcriptome. In the later stage of fruit development, PcWRKY11 can bind the W-BOX element in *PcPIF3* promoter of ‘Starkrimson’ pear instead of ‘Red Bartlett’ pear, leading to differences in anthocyanin accumulation patterns between the two cultivars.

## Materials and methods

### Plant materials and experimental treatments

All pear samples utilized in this study were collected from an orchard in Meixian, Shaanxi Province, China, in 2021. The ‘Starkrimson’ and ‘Red Bartlett’ were collected at 15, 30, 45, 60, 75 and 90 days after full bloom (DAFB). The ‘Red Sichou’ were bagged at 10 DAFB, and the bags were removed after 30 days of shading, prior to exposure to natural light. The faded fruits were used for transient silencing injection. Transient overexpression injection on ‘Zaosu’ pear at 40 DAFB, and these fruits were collected 5 days after injection. Fifteen fruits served as biological replicates, with three biological replicates for each time period. For further processing details, please refer to Cong et al. [39].

The UV intensity in the field was measured in Meixian. Starting from 15 DAFB to 105 DAFB, measurements were taken every 2 days at noon (12:00 PM) for the field's UV intensity. These measurements were averaged over each 15-day interval to calculate the daily average field UV intensity (μW/cm^2^) for six periods corresponding to different stages of pear fruit growth and development. Each measurement was replicated three times.

The isolated UV-B treatment experiment on fruits was conducted at the College of Horticulture in Northwest Agriculture and Forestry University. In a growth chamber equipped with UV-B lamps of varying radiation intensities, the fruits were subjected to 16 h of light exposure followed by 8 h of darkness at a temperature of 16 °C. The experiment utilized ‘Korla’ Pear sourced from the northern campus of Northwest Agriculture and Forestry University for UV-B radiation treatments of different intensities.

### Determination of anthocyanin content

Thoroughly grind the peel sample in liquid nitrogen, the extraction of anthocyanin was carried out according to the methods proposed by predecessors [40]. The gradient elution procedure was as reported earlier [41].

### RNA extraction and qRT-PCR

All samples were ground in liquid nitrogen and stored or used subsequently. For specific RNA extraction and reverse transcription methods, refer to Cong et al. [37].

All primers used in qreal-time PCR (qRT-PCR) were designed on NCBI web pages and were synthesized by AuGCT Biotech Company (Beijing, China). Primers are listed in Table S1. Detailed experimental methods and data processing can be found in Cong et al. [39]. All qRT-PCR reactions replicated three times for each biological repeat.

### Subcellular localization

The complete coding sequences (CDS) of *PcPIF3* and *PcWRKY11* were individually cloned into the plant binary expression vector pCambia 2300, which features the CaMV 35S promoter and a green fluorescent protein (GFP) tag, to form p35S::GFP-*PcPIF3* and p35S::GFP-*PcWRKY11*, resulting in fusion genes driven by the 35S promoter. The constructed plasmids were transformed into *Agrobacterium tumefaciens* strain EHA105 for *Nicotiana benthamiana* leaves. Inject the agrobacteria tumefaciens lines harbouring the vectors into *Nicotiana benthamiana* leaves. After injection, incubate in the dark for 1 d, and incubate under light at room temperature for 2 d, and then use an Olympus BX63 microscope (Olympus, Japan) to observe the *Nicotiana benthamiana* leaves. The primers were listed in Table S1.

### Transient expression assay in pear fruitlets skin

The complete coding sequence (CDS) of *PcPIF3* and the GUS gene were inserted into the multiple cloning site (MCS) of the pGreenII 0029 62-SK binary vector using *BamH*I and *Hind*III restriction enzymes [42]. The protocols for Agrobacterium culture and the injection of pear fruit were consistent with the previously described methods. The above two recombinant plasmids were respectively transformed into *Agrobacterium tumefaciens* strain EHA105, and the Agrobacterium was grown in a 28 °C incubator. The method of fruit injection referred to previous report [42]. Inject the activated Agrobacterium into the pear fruitlet peels, and collect them five days after the injection. The primers were listed in Table S1.

### Comparison of *PcPIF3* promoter activity between ‘Starkrimson’ pear and ‘Red Bartlett’ pear under different UV-B radiation intensities

The CDS of *GUS* was inserted into the multiple cloning sites (MCS) of the pGreenII 0029 62-SK binary vector with *BamH*I and *Hind*III [42]. The *PcPIF3* promoter sequences were cloned from the 5′ upstream region of the ATG start codon of *PcPIF3*. Promoter sequences with a length of 1856 bp were amplified from DNA of ‘Starkrimson’ pear and ‘Red Bartlett’ pear respectively. The promoters were cloned into the multiple cloning sites (MCS) of the pGreenII 0029 62-SK binary vector, replacing the original CaMV 35S promoter to drive GUS expression. The above two recombinant plasmids were respectively transformed into *Agrobacterium tumefaciens* strain EHA105, and the Agrobacterium was grown in a 28-degree incubator. The method of fruit injection referred to previous report [42]. Inject the activated Agrobacterium into the pear fruitlet peels, and the injected pears were placed in a fluorescent lamp and an ultraviolet light incubator with different UV-B intensities for cultivation. We divided the treatment under different UV intensities into 0, low, middle, high, corresponding to UV intensities of 0 μW/cm^2^, 30 μW/cm^2^, 60 μW/cm^2^ and 90 μW/cm^2^. The injected pears were collected three days later. The primers were listed in Table S1.

### Yeast one-hybrid assay

Y1H assays were conducted following the manufacturer's protocol, utilizing the Matchmaker Gold Yeast One-Hybrid System (Clontech, Mountain View, CA, USA). The 400 bp promoter fragment containing the core W-BOX 1 and 2 from the 'Starkrimson' pear peel and a 400 bp promoter fragment lacking the core W-BOX 1 and 2 from the 'Red Bartlett' pear peel were each inserted into the pAbAi vector. Then the CDS of *PcWRKY11* was cloned into the pGADT7 vector. Subsequent experimental methods were conducted according to Cong et al. [39]. The primers were listed in Table S1.

### Dual luciferase assay

In order to further screen the upstream candidate transcription factors, we performed a dual luciferase assay. The *PcPIF3* promoter fragments of ‘Starkrimson’ pear and ‘Red Bartlett’ pear were ligated into MCS of the reporter vector pGreen II 0800 LUC, and the CDS of *PcWRKY11* was cloned into the MCS of pGreenⅡ 0029-62SK vector to construct the effector plasmids. The primers were listed in Table S1. Subsequent experimental methods were conducted according to Cong et al. [37].

### GUS staining and quantitative GUS activity assay

The CDS of *PcWRKY11* were inserted into the plasmid pGreenII 0029 62-SK. The *PcPIF3* promoter sequence is cloned from the 5′ upstream region of the ATG start codon of *PcPIF3* around the W-BOX element. Promoter sequences with a length of about 200 bp were amplified from DNA of ‘Starkrimson’ pear and ‘Red Bartlett’ pear respectively. The promoters were inserted into the plasmid pCAMBIA1301-GUS and used as the reporter vector. GUS staining and Quantitative GUS activity assay refer to Blázquez [43].

### Electrophoretic mobility shift assay

The *PcWRKY11* and *PcPIF3* CDS were cloned into the pET-32a (+) vectors, respectively. The recombinant plasmids were inserted into *Escherichia coli* Rosetta (DE3) cells for the expression of proteins in a prokaryotic system. The *PcPIF3* promoter sequences were used to design the specific probes. The W-BOX 1 and W-BOX 2 site in 'Starkrimson' and its corresponding sequence in 'Red Bartlett', along with the 20 bp flanking regions, were used to design hot and cold probes. G-box on the *proPcMYB10* in 'Starkrimson' and 'Red Bartlett' along with the 20 bp flanking regions, were used to design hot, mutant and cold probes. Detailed experimental procedures follow those outlined in Sun et al. [44].

### Statistical analysis

Statistical analyses were performed using SPSS 22.0 software (SPSS, Chicago, IL, USA). A one-way ANOVA with Tukey’s honestly significant difference test was conducted to assess differences among means. GraphPad Prism 9.5 software (GraphPad Prism, San Diego, CA, USA) was utilized for figure creation. All experiments were conducted with three independent biological replicates. The significant difference was determined by one-way ANOVA for three replicates: *P < 0.05; **P < 0.01. Error bars represent the means ± SEM of biological replicates.

## Results

### Total anthocyanin accumulation and expression level analysis of PcPIF3 in two pear cultivars

**Fig. 1:**
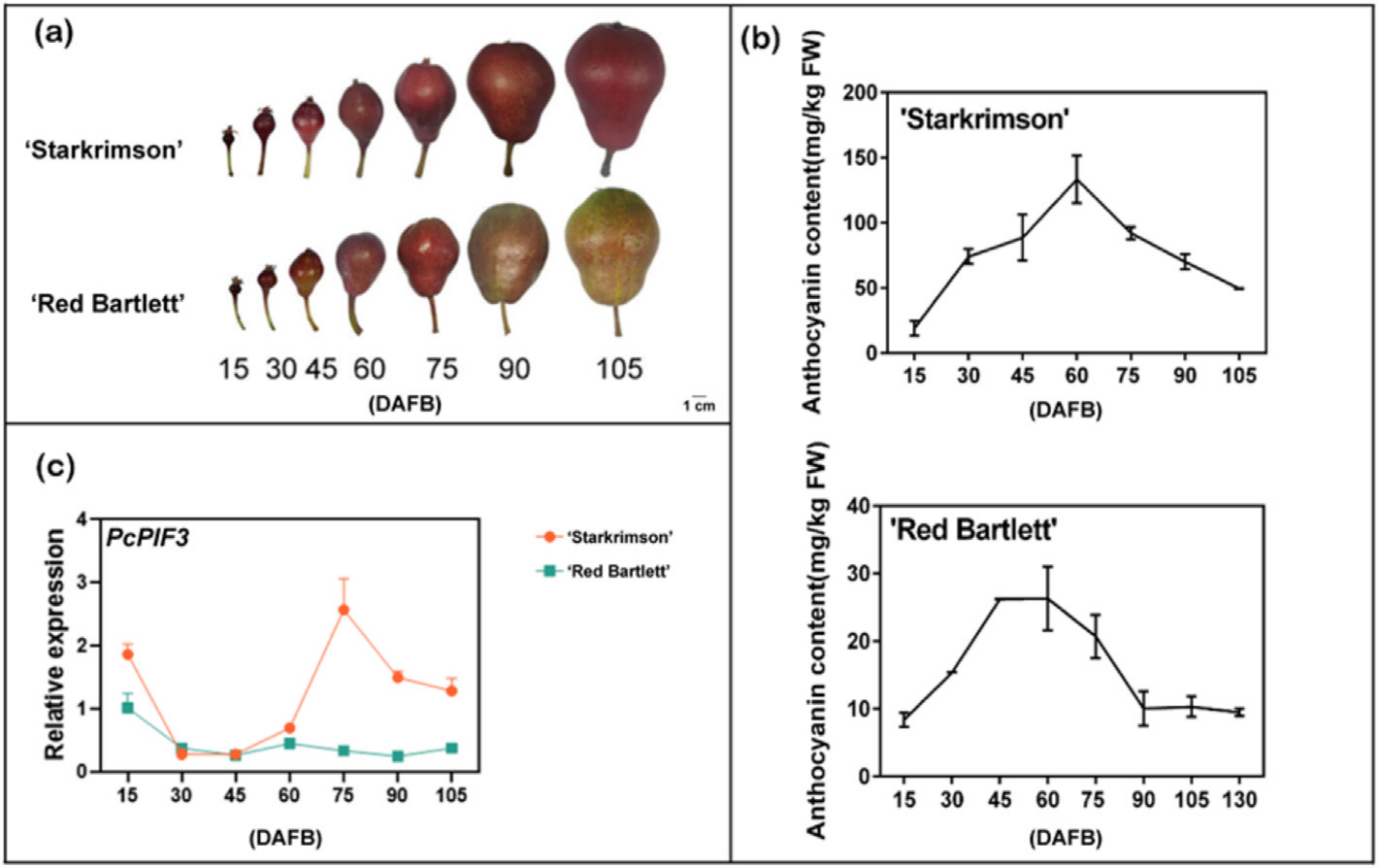
Changes in the expression of the *PcPIF3* gene and anthocyanin contents in two red pear cultivars at different stages of fruit development. (a) The phenotypes and changes in skin colour, along with anthocyanin contents, at various stages of ‘Starkrimson’ and ‘Red Bartlett’ pear development. (b) Total anthocyanin contents at different stages of fruit development. Data are presented as means ± standard deviations from three biological replicates. (c) Expression levels of the *PcPIF3* gene in the two red pear cultivars. (For interpretation of the references to color in this figure legend, the reader is referred to the web version of this article.)

‘Starkrimson’ pear and ‘Red Bartlett’ pear, both being red-skinned European pears, exhibit color fading during late fruit development, with ‘Red Bartlett’ showing more pronounced fading compared to “Starkrimson” (Fig. 1a). To investigate this phenomenon, we conducted seven samplings for each variety from 15 to 105 days after full bloom (DAFB) and assessed changes in anthocyanin content. Throughout the entire fruit development period, the anthocyanin levels in both ‘Starkrimson’ and ‘Red Bartlett’ pears exhibited a rise-and-fall trend (Fig. 1b). In the early stages of fruit development (from 15 to 60 days after full bloom, DAFB), the anthocyanin contents of both cultivars gradually increased, peaking at 60 DAFB. However, in the later stages of fruit development (from 75 to 105 DAFB), the anthocyanin levels began to decline. During this process, we found that the anthocyanin content of ‘Starkrimson’ pear continued to decrease in the late fruit development period (75 DAFB to 105 DAFB), while in ‘Red Bartlett’ pear, the anthocyanin content decreased in the 90 DAFB reaching the lowest value, close to none, and the anthocyanin content no longer decreased from 90 DAFB to 105 DAFB.

In previously, we found that the *PcPIF3* gene is related to the coloring of the pear peel [40]. Therefore, we analyzed the expression levels of the *PcPIF3* gene in the peels of both ‘Starkrimson’ pear and ‘Red Bartlett’ pear. The results indicated that during the early stages of fruit development (15 DAFB to 60 DAFB), when anthocyanin content was increasing, there was no significant difference in *PcPIF3* gene expression between the peels of ‘Starkrimson’ pear and ‘Red Bartlett’ pear. However, in the later stages of development (75 DAFB to 105 DAFB), as anthocyanin levels declined, the expression of *PcPIF3* in the peel of ‘Starkrimson’ pear was significantly higher than in ‘Red Bartlett’ pear (Fig. 1c). The expression level of *PcPIF3* exhibited a positive correlation with anthocyanin content changes between two pear varieties, suggesting its potential role in the colour fading phenomenon in pear peel. However, the precise mechanism driving this association remains unclear.

### Functional verification of PcPIF3

**Fig. 2:**
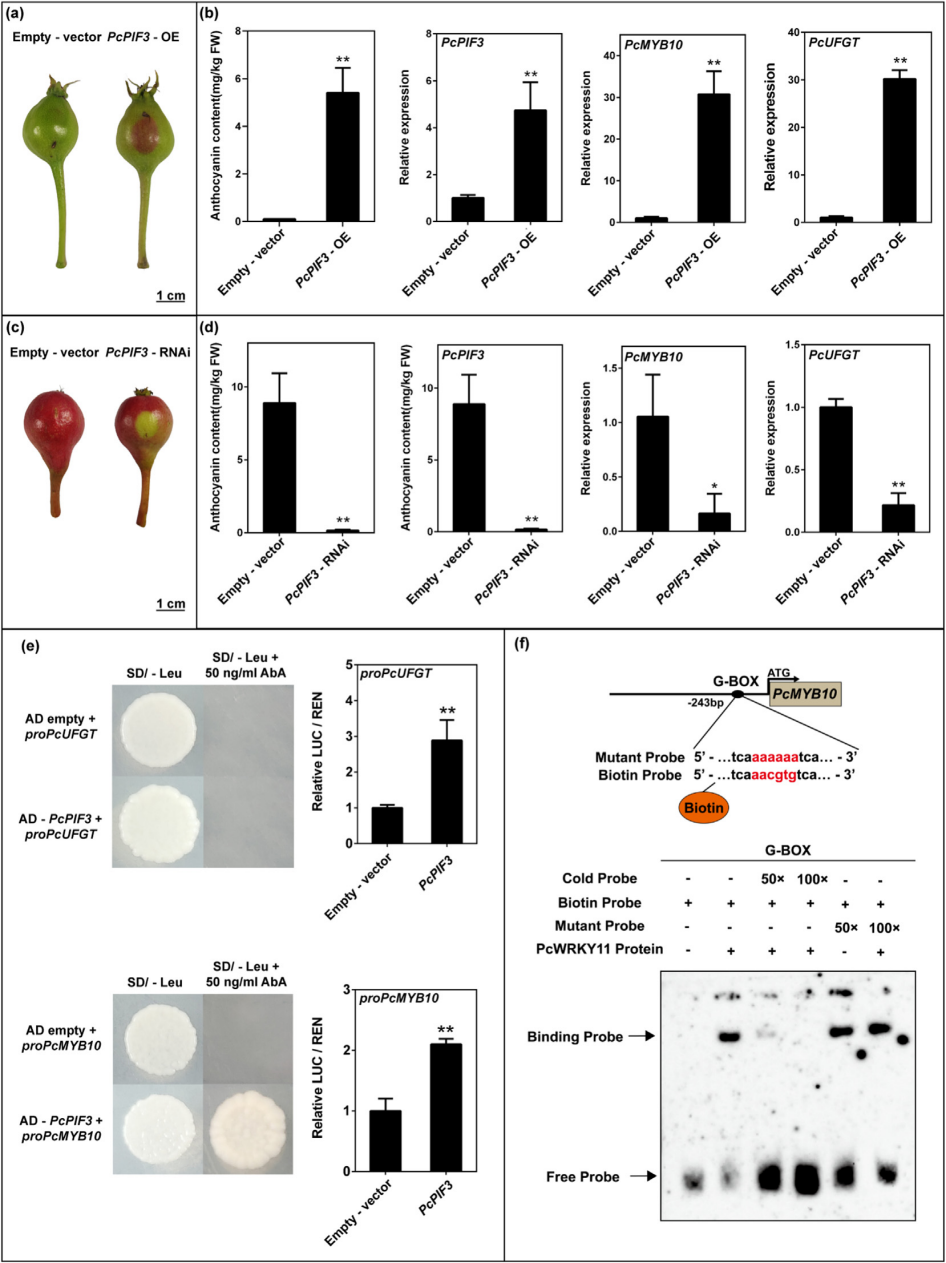
Transient transformation assays in the 'Zaosu' and 'Red Sichou' fruitlet peels to verify the function of *PcPIF3*. (a) The phenotype of 'Zaosu' fruitlet peels after transient overexpression of *PcPIF3*. (b) The anthocyanin contents and the expression levels of *PcPIF3*, *PcMYB10*, *PcUFGT* of ‘Zaosu’ fruitlet peels after transient overexpression of *PcPIF3*. (c) The phenotype of ‘Red Sichou’ fruitlet peels after transient *PcPIF3*-RNAi. (d) The anthocyanin contents and the expression levels of *PcPIF3*, *PcMYB10*, *PcUFGT* of ‘Red Sichou’ fruitlet peels after transient *PcPIF3*-RNAi. (e) The Y1H experiment revealed the relationship between PcPIF3 and the promoters of *PcMYB10* and *PcUFGT*. (f) EMSA experiments confirmed that PcPIF3 binds to the G-box site on the promoter region of PcMYB10. (For interpretation of the references to color in this figure legend, the reader is referred to the web version of this article.)

To investigate whether the *PcPIF3* gene influences anthocyanin accumulation in pear peel, we generated *PcPIF3*-OE and *PcPIF3*-TRV lines, and performed transient injections on the respective pear peel surfaces. In the peel of ‘Zaosu’ pear fruitlets, a transient overexpression assay of *PcPIF3* was performed using *Agrobacterium infiltration*, with the empty vector containing GUS used as the control. GUS staining results confirmed the feasibility of this transformation method (Supplemental Fig. S1). After three days of sunlight exposure, the peel of ‘Zaosu’ pear in the region injected with *PcPIF3*-OE turned red, in contrast to the area injected with the empty vector, which showed no colour change. (Fig. 2a). By measuring anthocyanin content in pear peel, we found that overexpression of *PcPIF3* significantly increased anthocyanin accumulation by increasing the transcription of *PcMYB10* and *PcUFGT*, which were key genes in the anthocyanin synthesis pathway (Fig. 2b). At the same time, we transiently silenced *PcPIF3* in the skin of the ‘Red Sichou’ pear fruitlets which was bagged to prevent fruit colouring before injection. After 5 days of transient transformation, the control area reverted to red under natural light, whereas the region injected with silenced *PcPIF3* failed to regain red coloration (Fig. 2c). Compared to the control, silencing *PcPIF3* significantly reduced anthocyanin biosynthesis in pear peel. Additionally, the expression levels of key genes in the anthocyanin synthesis pathway, *PcMYB10* and *PcUFGT,* were markedly downregulated (Fig. 2d).

The dual luciferase assay and Y1H experiments confirmed that PcPIF3 binds to the promoter regions of *PcMYB10* but not *PcUFGT*, thereby enhancing their transcription (Fig. 2e). Further EMSA experiments confirmed that PcPIF3 binds to the G-box site in the promoter region of *PcMYB10* (Fig. 2f). Additionally, subcellular localization experiments revealed that PcPIF3 is located in the nucleus (Supplemental Fig. S2).

The results indicate that the *PcPIF3* gene plays a crucial role in regulating anthocyanin accumulation in red pear fruits. However, the mechanism by which it influences the difference in anthocyanin content during the ripening process between 'Red Bartlett' and 'Starkrimson' pears remains to be further investigated.

### Strong UV-B radiation activates the PcPIF3 promoter of ‘Starkrimson’ pear instead of the promoter of ‘Red Bartlett’ pear

In previous study, the expression levels of *PcPIF3* in 'Starkrimson' and 'Red Bartlett' pears were similar during the early stages of fruit development (before 60 DAFB). However, a significant difference emerged in the later stages (75 DAFB to 105 DAFB) (Fig. 1c). Previous studies have indicated that PIF3 plays a crucial role in UV-B-induced anthocyanin biosynthesis. Furthermore, by monitoring the UV-B radiation intensity throughout the entire developmental period of pears, it was observed that the UV-B radiation intensity was significantly higher during the late stages of fruit development compared to the early stages (Supplemental Fig. S3).To validate this assumption, UV-B treatment with varying intensities was applied to pear fruits. The results confirmed the assumption at various levels, including phenotypic, anthocyanin content, and gene expression levels (Supplemental Fig. S4). Therefore, we speculate that the difference in the expression of *PcPIF3* in the later stage is related to the UV-B radiation intensity. Then, through indoor simulation experiments, we analysed the relationship between the UV-B radiation intensity and the *PcPIF3* promoters of ‘Starkrimson’ pear and ‘Red Bartlett’ pear respectively.

**Fig. 3:**
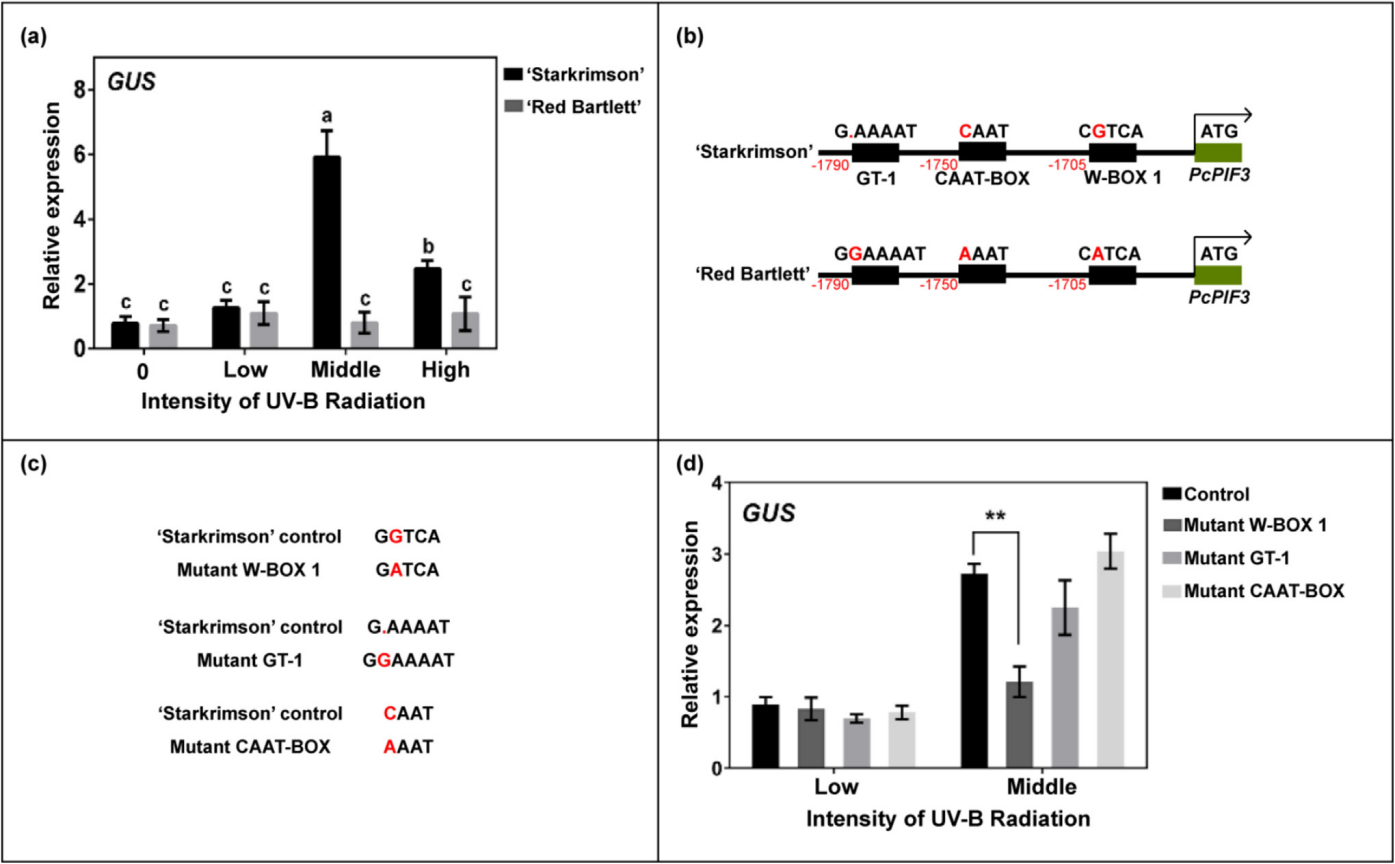
Analysis of promoter activity of PcPIF3 under different UV-B radiation intensity. (a) The promoter activity of PcPIF3 in ‘Starkrimson’ pear and ‘Red Bartlett’ pear under different UV-B radiation intensity. (b) Nucleotide sequence of PcPIF3 promoter of ‘Starkrimson’ pear and ‘Red Bartlett’ pear. (c) Three site mutations in the *cis*-acting elements of the PcPIF3 promoter in ‘Starkrimson’ pear. (d) Promoter activity of PcPIF3 promoter element point mutations under different UV-B radiation intensities. (For interpretation of the references to colour in this figure legend, the reader is referred to the web version of this article.)

We cloned the promoter regions of ‘Starkrimson’ pear and ‘Red Bartlett’ pear, and subsequently constructed the 62SK-*proPcPIF3*-GUS vectors for each promoter. The two vectors will be injected into the pear peel by transient Agrobacterium transformation. The injected pears were treated with different UV-B radiation intensities. The results showed that the *PcPIF3* promoter of ‘Starkrimson’ pear responded to the regulation of downstream genes with high UV-B radiation intensity, which was strongest in middle UV-B radiation intensity. On the contrary, the regulation of downstream genes by the *PcPIF3* promoter in the ‘Red Bartlett’ pear was not affected by UV-B radiation (Fig. 3a).

In order to further explore the regulation path of UV-B on *PcPIF3*, we compared the *PcPIF3* promoter sequences on the peel of ‘Starkrimson’ pear and ‘Red Bartlett’ pear. The results revealed that there are indeed differences in the nucleotide sequences of the *PcPIF3* promoter between ‘Starkrimson’ pear and ‘Red Bartlett’ pear, and the specific base differences are shown in the Fig. 3b. The two promoter fragments were analysed by the web of New Place, and three possible different elements, namely ‘W-BOX 1’, ‘GT-1’ and ‘CAAT-BOX’ were found. Among them, the ‘W-BOX’ was at position −1705, the ‘CAAT-BOX’ was at position −1750 and the ‘GT-1’ was at position −1790, Where 'GT-1′ is deleted, and ‘W-box 1’ and ‘CAAT-box’ are substituted. (Fig. 3b).

In order to verify these different elements, a site-directed mutagenesis test was conducted (Fig. 3c), and three constructed vectors 62SK*-proPcPIF3*-GUS that do not contain ‘W-BOX 1’, ‘GT-1’ and ‘CAAT-BOX’ were obtained respectively. The Agrobacterium carrying these three vectors was injected into the pear peel, and the unmutated ‘Starkrimson’ pear 62SK*-proPcPIF3*-GUS was used as a control. Under low UV-B radiation intensity, the activities of the three mutant ‘Starkrimson’ pear *proPcPIF3* were not significantly different from the control under moderate UV-B radiation intensity. The ‘Starkrimson’ pear *proPcPIF3* activity without ‘W-BOX 1’ significantly lowered than the control (Fig. 3d). This indicates that UV-B may regulate *PcPIF3* through the W-BOX element on the *PcPIF3* promoter of ‘Starkrimson’ pear. This suggests that there may be upstream genes binding to this site, affecting the transcription of *PcPIF3*.

### Upstream transcriptional regulation of PcPIF3 gene

**Fig. 4:**
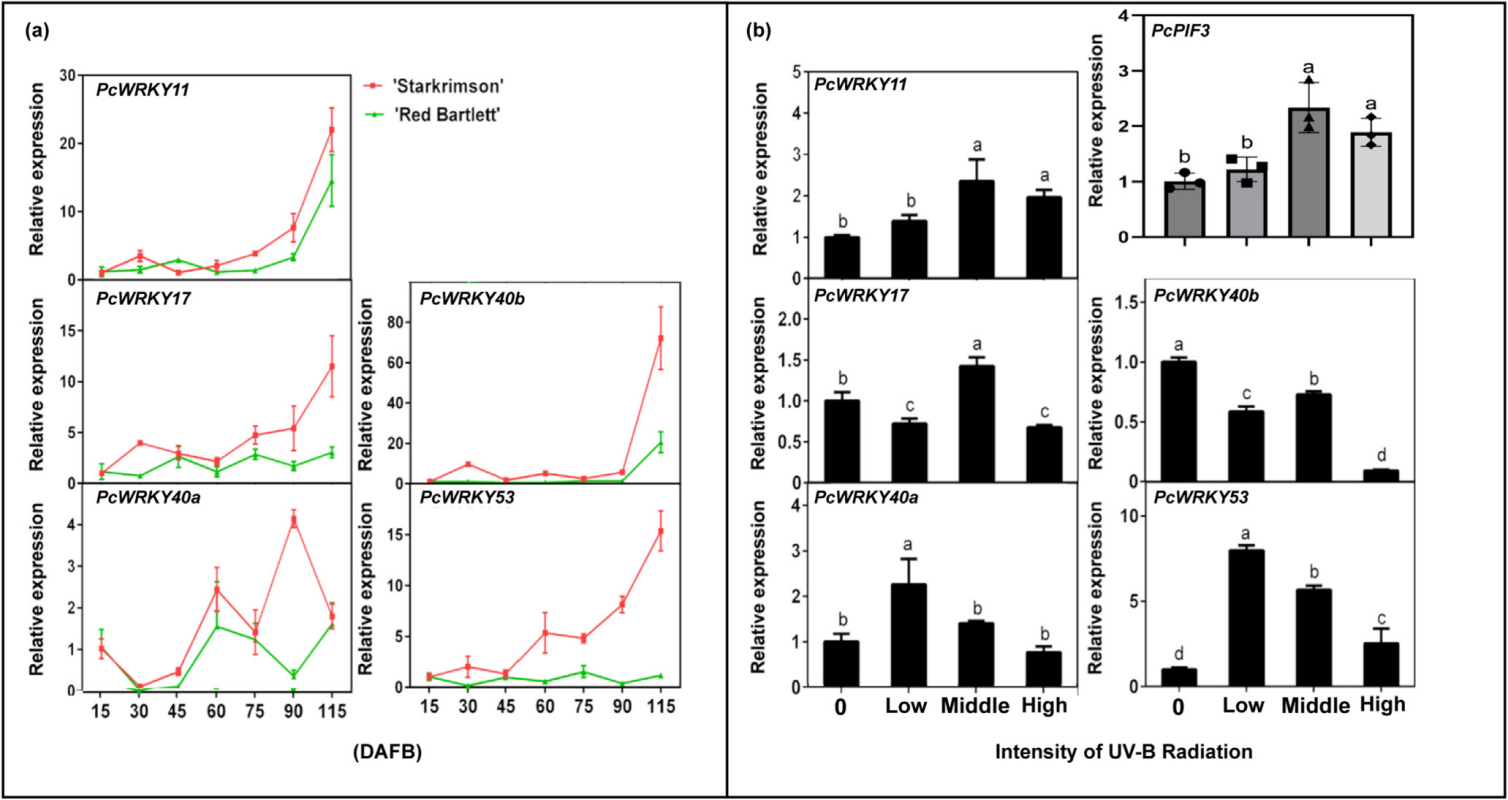
Screening and validation of upstream regulators for the *PcPIF3* gene. (a)The expression levels of candidate upstream regulators *PcWRKY11*, *PcWRKY17*, *PcWRKY40a*, *PcWRKY40b* and *PcWRKY53* at different stages of ‘Starkrimson’ pear and ‘Red Bartlett’ pear development. (b) The expression levels of candidate upstream regulators *PcPIF3*, *PcWRKY11*, *PcWRKY17*, *PcWRKY40a*, *PcWRKY40b* and *PcWRKY53* under different UV-B radiation intensity. (For interpretation of the references to color in this figure legend, the reader is referred to the web version of this article.)

*WRKY* gene family members are known as potential upstream transcription factors that can bind to W-box elements. By analysing the differentially expressed genes in transcriptomes of the peels of the 75 DAFB ‘Starkrimson’ pear and ‘Red Bartlett’ pear, five *WRKY* family genes were screened out which can conservedly bind to the W-BOX 1 (Supplemental Table S2). The transcription abundance of these *WRKY* family genes on the peel of ‘Starkrimson’ pear was higher than that of ‘Red Bartlett’ pear. Expression analysis of the *WRKY* family genes was conducted in the peels of ‘Starkrimson’ pear and ‘Red Bartlett’ pear at various developmental stages. The expression of *PcWRKY40a* showed irregular patterns throughout fruit development. In the early stages for both pear cultivars, the expression levels of *PcWRKY11* and *PcWRKY40b* were lower under low-intensity UV-B radiation. Under higher UV-B radiation intensity, the expression levels of *PcWRKY11* and *PcWRKY40b* increased significantly during the later stages. The expression levels of *PcWRKY17* and *PcWRKY53* showed an upward trend during the fruit development of ‘Starkrimson’ pear, while the expression of *PcWRKY17* and *PcWRKY53* continued to be low-level during fruit development of ‘Red Bartlett’ pear. This result suggests that the expression of *PcWRKY11* and *PcWRKY40b* might be related to the UV-B radiation intensity, but the expression of *PcWRKY17* and *PcWRKY53* had no correlation with UV-B radiation intensity (Fig. 4a).

In order to further verify the response of several genes to UV-B radiation intensity, the ‘Zaosu’ pears were irradiated with different intensities of UV-B for 3 days, we found that after high UV-B radiation treatment, the expression of *PcWRKY11* in the pear peel has been significantly increased (Fig. 4b), and it is consistent with the change trend of the ‘Starkrimson’ pear promoter activity under different UV-B radiation intensities (Fig. 3a). On the contrary, the expression trend of *PcWRKY17*, *PcWRKY40a*, *PcWRKY40b* and *PcWRKY53* is not consistent with the above (Fig. 4b). Simultaneously, the expression level of *PcWRKY11* showed similar variations under different UV-B radiation intensities as *PcPIF3* (Fig. 4b). This confirms that PcWRKY11 is a potential upstream transcription factor that regulates *PcPIF3*.

### Transient assay of PcWRKY11 in pear fruitlet peel

**Fig. 5:**
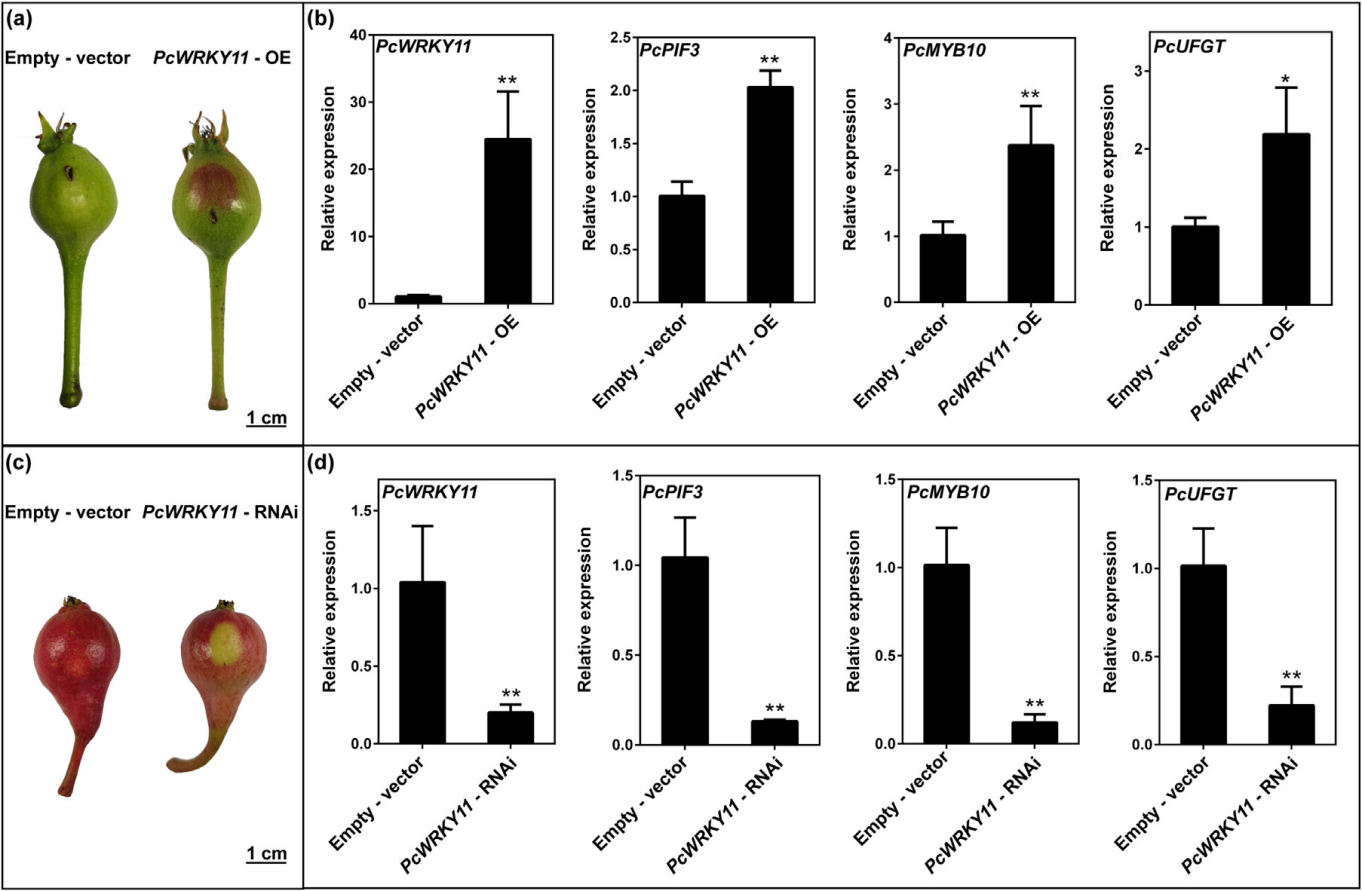
Transient transformation assays in the ‘Zaosu’ and ‘Red Sichou’ fruitlet peels to verify the function of *PcWRKY11*. (a) The phenotype of ‘Zaosu’ fruitlet peels after transient overexpression of *PcWRKY11*. (b) The anthocyanin contents of ‘Zaosu’ fruitlet peels after transient overexpression of *PcWRKY11* and the expression levels of *PcWRKY11*, *PcPIF3*, *PcMYB10*, *PcUFGT* of ‘Zaosu’ fruitlet peels after transient overexpression of *PcWRKY11*. (c) The phenotype of ‘Red Sichou’ fruitlet peels after transient *PcWRKY11*-RNAi. (d) The anthocyanin contents of ‘Red Sichou’ fruitlet peels after transient *PcWRKY11*-RNAi and the expression levels of *PcWRKY11*, *PcPIF3*, *PcMYB10*, *PcUFGT* of ‘Red Sichou’ fruitlet peels after transient *PcWRKY11*. (For interpretation of the references to color in this figure legend, the reader is referred to the web version of this article.)

To further investigate the role of *PcWRKY11* in regulating anthocyanin accumulation, a transient transformation experiment was performed on pear peels in a field setting. After *PcWRKY11* was overexpressed on the skin of ‘Zaosu’ pear young fruit, the injection area turned red (Fig. 5a), the anthocyanin content was significantly increased (Fig. 5b), and the expression of *PcMYB10* and *PcUFGT* were significantly upregulated following the transient transformation (Fig. 5b).

After silencing *PcWRKY11* on the skin of young ‘Red Sichou’ fruit, the injected area did not return to red (Fig. 5c). Compared with the control, anthocyanin content was significantly reduced (Fig. 5d), along with a notable decrease in the expression of key genes *PcMYB10* and *PcUFGT* in the anthocyanin biosynthesis pathway (Fig. 5d). This suggests that *PcWRKY11* positively regulates anthocyanin synthesis. After overexpression of *PcWRKY11*, the expression of *PcPIF3* was significantly increased (Fig. 5b), and after silencing *PcWRKY11*, the expression of *PcPIF3* was significantly reduced (Fig. 5d), which indicates that *PcWRKY11* may have a regulatory effect on the expression of *PcPIF3*. In the 120 DAFB fruits of ‘Red Anjou’ skin, transient overexpression of *PcWRKY11*-OE also yielded similar results (Supplemental Fig. S5).

Given that PcWRKY11 can also promote *PcPIF3* expression in ‘Red Anjou’ and ‘Zaosu’, we suspect the existence of more ubiquitous sites enabling WRKY11 regulation of *PIF3* expression in pears. To this end, the promoter sequences of *PIF3* from multiple pear varieties were cloned and sequenced. Through comparison, it was found that the promoter regions of *PIF3* genes in pears with different genetic backgrounds contain distinct W-BOX elements, which are potential binding sites for WRKY11 (Supplemental Fig. S6).

To determine the specific location of the PcWRKY11 protein, the Subcellular localization experiments was carried out. The results showed PcWRKY11 was located in the nucleus (Supplemental Fig. S2).

### PcWRKY11 binds to the PcPIF3 promoter of ‘Starkrimson’ pear at the specific W-BOX element

**Fig. 6:**
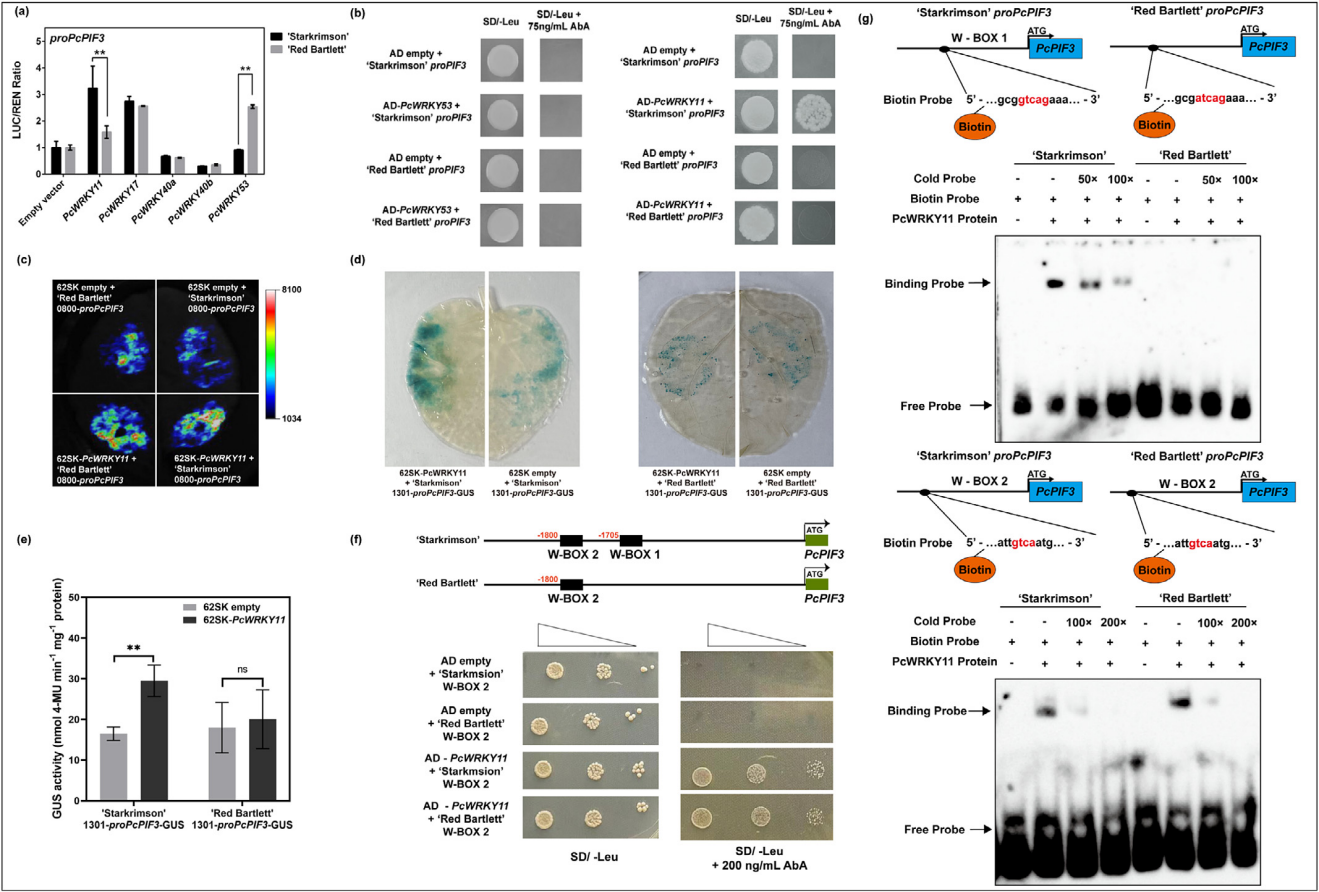
PcWRKY11 binds to the *PcPIF3* promoter of ‘Starkrimson’ pear (a) Effects of candidate upstream regulators PcWRKY11, PcWRKY17, PcWRKY40a, PcWRKY40b and PcWRKY53 on the promoter activity of *PcPIF3* in two red pear cultivars in a dual luciferase assay. (b) In Y1H assays, the interaction of PcWRKY11 and PcWRKY53 with the *PcPIF3* promoters in two red pear cultivars. (c) LUC experiment showing the binding of PcWRKY11 to the ‘Starkrimson’ *proPcPIF3* in vivo. Agroinfiltrated *Nicotiana benthamiana* leaves were analyzed at 3 days. (d) GUS staining and (e) GUS enzyme activity confirmed the binding of PcWRKY11 to the W-BOX element in ‘Starkrimson’ *proPcPIF3*. (f) The Y1H experiment demonstrates that PcWRKY11 can bind to the W-BOX 2 element in both ‘Starkrimson’ and ‘Red Bartlett’. (g) The EMSA experiment confirmed that the PcWRKY11 protein can bind to the W-BOX 1 site in ‘Starkrimson’ but not in ‘Red Bartlett’, binding to the W-BOX 2 site in ‘Starkrimson’ and ‘Red Bartlett’. (For interpretation of the references to color in this figure legend, the reader is referred to the web version of this article.)

Given that *PcPIF3* and *PcWRKY11* exhibit similar expression patterns under UV-B treatment, we suspect they may have a potential regulatory relationship. To further confirm the interaction between *PcWRKY11* and *PcPIF3* in the peels of ‘Starkrimson’ pear and ‘Red Bartlett’ pear, a dual-luciferase assay was performed. The results demonstrated that PcWRKY11 exhibited an activating effect on the *PcPIF3* promoter in ‘Starkrimson’ pear. (Fig. 6a). Then, to further confirm the interaction between PcWRKY11 and the W-BOX 1 element in the peel of both ‘Starkrimson’ and ‘Red Bartlett’ pears, Y1H experiments were conducted. Based on the promoter divergence analysis shown in Fig. 3, we preliminarily identified W-box 1 as a potential binding site for Y1H experiments. The results show that PcWRKY11 can bind to the promoter of *PcPIF3* in ‘Starkrimson’ pear which contains the core W-BOX 1 but cannot bind to the promoter of *PcPIF3* ‘Red Bartlett’ pear which doesn’t contain the core W-BOX 1 (Fig. 6b). To validate the results of Fig. 6a & b, we further confirmed that PcWRKY11 specifically regulates the ‘Starkrimson’ *PcPIF3* promoter in vivo through the LUC experiment. The results demonstrate that PcWRKY11 can only bind to the ‘Starkrimson’ *proPcPIF3*, not the ‘Red Bartlett’ (Fig. 6c). To ascertain whether PcWRKY11 can enhance its expression by binding to the promoter of *PcPIF3*, GUS staining and enzyme activity assays were conducted to analyze its promoter activity. The results revealed that the PcWRKY11 + ‘Starkrimson’ *proPcPIF3* group exhibited deeper coloration compared to the other groups (Fig. 6d), with GUS enzyme activity also higher than the other groups (Fig. 6e).

Due to the insignificant increase in GUS enzyme activity in the experimental group compared to the control group in 'Red Bartlett', there is a suspicion whether PcWRKY11 might bind to other locations in the *PcPIF3* promoter region to activate its expression (Supplemental Fig. S6). Through promoter region analysis, it was discovered that in ‘Starkrimson’, there is another W-BOX element, named W-BOX 2, shared with 'Red Bartlett', located before the mutated W-BOX site. Through Y1H assay validation, it was confirmed that PcWRKY11 can bind to this W-BOX 2 in both ‘Starkrimson’ and ‘Red Bartlett’ (Fig. 6f). The results above indicate that PcWRKY11 enhances PcPIF3 expression by binding to the additional W-BOX 1 element introduced by a mutation in the 'Starkrimson' *PcPIF3* promoter region.

Given that the previous experiments only demonstrated that PcWRKY11 can bind to the W-BOX 1 site on the ‘Starkrimson’ *PcPIF3* promoter and activate it in vivo, but did not reveal their relationship in vitro, EMSA can provide further evidence of protein-DNA interaction outside the cell. By constructing biotin-labelled probes and corresponding cold probes based on the sequences near the W-BOX 1 region of ‘Starkrimson’ and ‘Red Bartlett’ the ability of these probes to specifically bind PcWRKY11 protein was compared. The results showed that only the lane corresponding to the ‘Starkrimson’ probe displayed a specific binding band, while no band was observed for the ‘Red Bartlett’ probe (Fig. 6g). This provides further evidence that the PcWRKY11 protein can specifically bind to the W-BOX 1 site of the ‘Starkrimson’ *PcPIF3* promoter in vitro as well.

## Discussion

The vibrant red colour of European red-skinned pears diminishes significantly during the later stages of fruit development due to a reduction in anthocyanin content, adversely affecting both the fruit's nutritional and economic value. Thus, it is crucial to explore the mechanisms underlying anthocyanin accumulation in red-skinned European pears. The ‘Starkrimson’ variety, which demonstrates higher anthocyanin levels in the later stages of development compared to other red-skinned European pear cultivars, experiences less pronounced colour fading. So, through transcriptome screening, we identified a differentially expressed gene, *PcPIF3*, and confirmed its involvement in regulating anthocyanin synthesis. Subsequently, we uncovered the transcription factor PcWRKY11, which promotes *PcPIF3* gene expression under UV-B. We further determined that the differential sequence in the W-BOX 1 of *proPcPIF3* under UV-B in the PcWRKY11-PcPIF3 module is the cause of the variation in anthocyanin accumulation in pear skin. In summary, we investigated the regulatory mechanism of the *PcWRKY11-PcPIF3* module under UV-B, leading to differences in anthocyanin synthesis in red-skinned pears.

Light is a critical environmental factor for the growth and development of most plants, engaging in physiological and reproductive growth processes through PIFs [45], various types of light affect PIFs differently in terms of mechanisms and outcomes. In Arabidopsis, UV-B-induced AtPIF4/5 promotes hypocotyl elongation [46]. UVR8, dependent on UV-B, destabilizes PIF1 protein through COP5, thus suppressing stem elongation in response to sunlight [47]. Recent studies have even suggested that UV-B-dependent CsPIF3 can integrate red/far-red and UV-B light signalling pathways to influence downstream gibberellin and auxin-dependent cucumber hypocotyl growth [48]. Similarly, blue or red light influences the expression of PIFs through CRY1/2 [24,49] and phyA/B [50,51], leading to subsequent changes in gene expression and phenotypes. This alteration encompasses the accumulation of anthocyanins [50]. While it has been reported that UV-B affects the accumulation of anthocyanins in plants [15], the emphasis has been placed on the regulation of structural and regulatory genes involved in anthocyanin synthesis by the UVR8-HY5 pathway [52]. The role of PIFs in this pathway remains unexplored [53]. In this study, we primarily focused on the reasons for the coloration differences during the later stages of fruit development, considering the significant increase in UV-B intensity in the field during this period as a key environmental factor. Our study revealed that UV-B significantly upregulates the expression of *PcPIF3*, thereby affecting the accumulation of anthocyanins (Fig. 3). Interestingly, it was observed that excessively high levels of UV-B treatment do not further increase the expression of *PcPIF3*. This suggests that only moderate UV-B intensity might stimulate *PcPIF3* expression (Supplemental Fig. S4), and *PcPIF3* is likely a key mediator in the response of European pear to low and moderate UV-B intensity. High-intensity UV-B radiation can be lethal to plants under such conditions, leading to the cessation of various biological processes, including anthocyanin biosynthesis. We have reported another HY5-independent pathway through which downstream gene respond to UV-B, providing a new potential mechanism for light involvement in plant physiological processes through *PIFs*.

The functions and mechanisms through which PIFs regulate anthocyanin biosynthesis in plants are complex. AtPIF3 can moderately suppress anthocyanin accumulation in response to NaCl, low nitrogen (−N), or 6-benzylaminopurine (6-BA) treatments [54], while it promotes anthocyanin biosynthesis under normal light conditions in a HY5-dependent manner in Arabidopsis [30,31]. Existing research suggests that PIFs can be involved in anthocyanin biosynthesis through mechanisms involving protein–protein interactions or by binding to the promoter regions of downstream genes [48,55]. For instance, MYB30 interacts with PIF3 to regulate photomorphogenic development in Arabidopsis [56], CsPIF3 inhibits the flavonoid pathway by activating *MYB7* in tea plants [57]. Similarly, under white light, PIF4 directly binds to the W-BOX element on the promoter of *AtPAP1* (also known as *AtMYB75*) to inhibit anthocyanin accumulation in Arabidopsis [58]. Different PIFs have varying effects on anthocyanin biosynthesis in pears. PyPIF5 binds to the *miR156b* promoter region to inhibit anthocyanin biosynthesis [33], while PpPIF8 enhances anthocyanin accumulation by stimulating the transcriptional activity of the anthocyanin biosynthesis structural gene *PpCHS* [34]. In this study, PcPIF3 simultaneously activated the transcriptional activity of both structural and regulatory genes involved in anthocyanin biosynthesis, thereby promoting anthocyanin accumulation (Fig. 2 & Supplemental Fig. S2). This provides insights for exploring additional functions of PIFs in anthocyanin regulation.

WRKY transcription factors influence the transcriptional activity of downstream genes and are involved in nearly all aspects of plant life activities. WRKY transcription factors modulate anthocyanin biosynthesis by binding to W-BOX elements in the promoter regions of downstream genes associated with anthocyanin biosynthesis, thereby influencing the expression levels of these target genes [3,38,39]. In this study, we found that PcWRKY11 also binds to the W-BOX elements in the promoter region of *PcPIF3* in ‘Starkrimson’ pears and enhances the expression of *PcPIF3* under UV-B exposure (Fig. 3, Fig. 6). Given that other WRKY family members (such as *PcWRKY40a* and *PcWRKY53*) also show high gene expression levels in response to UV-B or during fruit development, they are likely involved in the regulation of anthocyanin accumulation under UV-B as well. However, since *PcWRKY11* exhibits the expected patterns in both UV-B responsiveness and expression levels, and its expression pattern shows a very high similarity after UV-B treatment with *PcPIF3*, this study primarily focuses on *PcWRKY11*. But we also found that the expression level of *PcWRKY11* is similar to *PcPIF3*, and under high UV-B intensity, it did not show significant differences compared to medium UV-B intensity. This suggests that we cannot rule out a negative regulatory mechanism that inhibits the overactivation of *PcWRKY11* under high UV-B conditions. Previous studies have reported that under UV-B exposure, the expression of the *OsWRKY89* gene in rice is enhanced in response to abiotic stress [59], but it remains unknown whether this pathway involves UVR8 participation, similar to the AtUVR8-AtWRKY36 interaction observed in Arabidopsis [60]. This provides direction for exploring how transcription factors respond to UV-B and subsequently influence downstream pathways. In our next step, we will focus on investigating how UV-B affects the expression of *PcWRKY11*.

**Fig. 7:**
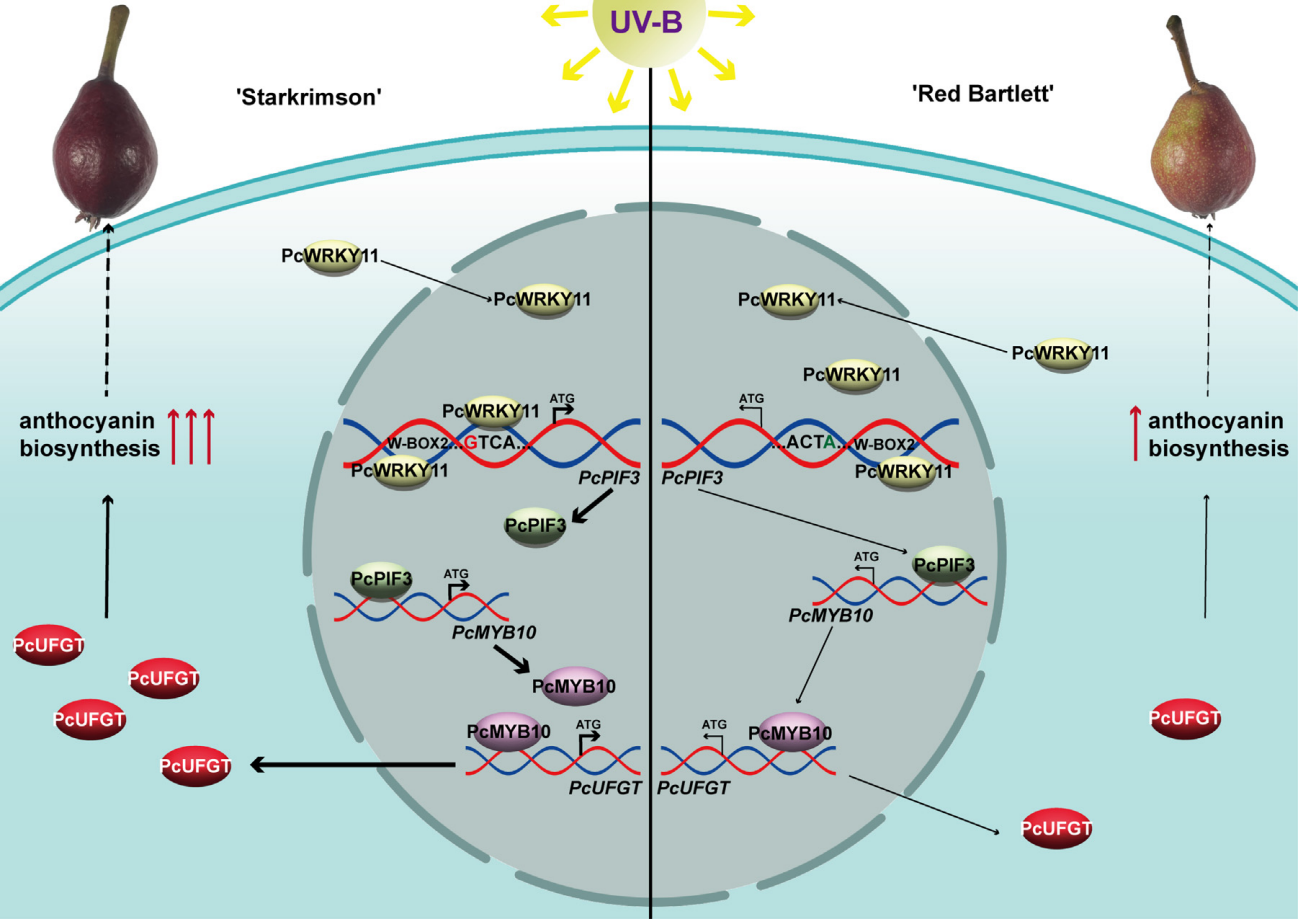
Model of *PcPIF3* Response to UV-B and Its Impact on Anthocyanin Accumulation in Pear Fruit Skin. Under UV-B irradiation, *PcPIF3* is activated, influencing the expression of *PcUFGT* and *PcMYB10*, thereby promoting anthocyanin biosynthesis. Differential W-BOX 1 motifs in the *proPcPIF3* region between 'Red Bartlett' and 'Starkrimson' lead to selective binding of PcWRKY11 to the *proPcPIF3* W-BOX 1 in 'Starkrimson', enhancing expression. This discrepancy in anthocyanin content consequently affects pear skin coloration. (For interpretation of the references to color in this figure legend, the reader is referred to the web version of this article.)

'Starkrimson' and 'Red Bartlett' pear skins both experience discoloration during late fruit development, but the degree of discoloration varies [61]. The difference in this discoloration could be attributed to genetic variations. In this study, we have demonstrated that the mutations in the W-BOX 1 elements in the 'Starkrimson' *PcPIF3* promoter region allows PcWRKY11 to bind to the specific *PcPIF3* promoter region of ‘Starkrimson’ pear but not ‘Red Bartlett’. Under UV-B exposure, the ‘Starkrimson’ *PcWRKY11-PcPIF3* module is enhanced, leading to increased expression of *PcMYB10* and *PcUFGT* mediated by *PcPIF3*, resulting in higher anthocyanin accumulation. On the other hand, the PcWRKY11 of ‘Red Bartlett’ cannot bind to the specific *PcPIF3* promoter region, which leads to lower anthocyanin accumulation compared to ‘Starkrimson’ pear. This results in differences in the extent of red color fading in ‘Starkrimson’ and ‘Red Bartlett’ pears during the late stages of fruit development, as the field UV-B intensity increases (Fig. 7). This increase in upstream protein binding sites caused by such mutations is likely a means of quantitatively measuring plant sensitivity to certain abiotic stresses.

## Conclusion

This study initially identified a potential relationship between *PcPIF3* and anthocyanin accumulation in red-skinned European pear. After validating its role in promoting anthocyanin accumulation, we further investigated the differences in the promoter region of *PcPIF3* between ‘Red Bartlett’ and ‘Starkrimson’ pears. Therefore, we have discovered that under UV-B, PcWRKY11 binds to the specific site of the W-BOX 1 element in ‘Starkrimson’, affecting the transcription of *PcPIF3*, thereby resulting in differences in anthocyanin biosynthesis between the two red-skinned pear varieties.

## CRediT authorship contribution statement

**Haowei Cao:** Methodology, Investigation, Formal analysis, Writing – original draft. **Yingying Qu:** Methodology, Investigation, Formal analysis, Writing – original draft. **Lei Guo:** Validation, Formal analysis, Visualization. **Mengjia Wu:** Formal analysis, Visualization. **Guorong Zhang:** Resources, Writing – review & editing. **Ying Tang:** Resources, Writing – review & editing. **Hongjuan Zhang:** Validation, Visualization. **Rui Zhai:** Validation, Data curation. **Chengquan Yang:** Methodology, Resources. **Lingfei Xu:** Methodology, Validation, Project administration, Conceptualization, Writing – review & editing, Supervision. **Zhigang Wang:** Methodology, Validation, Project administration, Conceptualization, Writing – review & editing, Supervision.

## Funding

This research was funded by the National Natural Science Foundation of China (No. 31972372) and Weinan Experimental Station Special Project (2024WNXNZX-4).

## Declaration of competing interest

The authors declare that they have no known competing financial interests or personal relationships that could have appeared to influence the work reported in this paper.
