# Supplementary material for: Anthocyanin accumulation differences in European pears caused by Phytochrome-interacting factor 3 (PcPIF3) promoter mutations under UV-B
Source: J Adv Res. 2025 May 13;80:125–36. doi: 10.1016/j.jare.2025.05.010 (PMC12869242; doi:10.1016/j.jare.2025.05.010)
Supplement: Supplementary Data 1 [file mmc1.pdf]

SUPPLEMENTAL DATA

Supplemental Table S1. Primers used in the experiment.

qRT - PcPIF3-F	CTTACCGTCTTTTAGCAACCCA
qRT - PcPIF3-R	AGAAGATGGCACTGACAACG
qRT - PcActin-F	ACAGTGTCTGGATTGGAGGGTC
qRT - PcActin-R	CATTTGGAGAACTCAGAAGCACT
qRT - PcUFGT-F	TACCAAATGGGACAAACGCTTC
qRT - PcUFGT-R	TCCAGCCACTCTAAGCAACCAC
qRT - PcMYB10-F	AGGGCTGCATGTCCCAGC
qRT - PcMYB10-R	CATGCCACATTTACAAGCAAGG
qRT - PcWRKY40a-F	AGGTCACCAAAGACAACCCTT
qRT - PcWRKY40a-R	CGCCACGAGGATGGAATCAT
qRT - PcWRKY53-F	AGATGCACGTACCGGAACAC
qRT - PcWRKY53-R	AACTGCCGCCATGAGAACAT
qRT - PcWRKY40b-F	CTCTTTTGCTCCAAGCTGCC
qRT - PcWRKY40b-R	ACCGATCCTATGGTCATGCG
qRT - PcWRKY11-F	TCATCGATTTCCGTGCCTCC
qRT - PcWRKY11-R	CAGTGGCCAGACGATGATGT
qRT - PcWRKY17-F	GTGACTGTTCCGGCTATCGT
qRT - PcWRKY17-R	ACACTGAAGCTCTCCTTCGC
PcPIF3-OE-F	ATGCCTTTGTCCGAGCTTTAT
PcPIF3-OE-R	CTATCCGTCAGCTCTGTTGTTGT
PcPIF3 - RNAi-F	TGCAAATGAGGCTTCCACC
PcPIF3 - RNAi-R	CACTACTGCTGGAATCAATTAGCAT
PcWRKY11-OE-F	ATGGCTGTAGATCTAGTTGGCTTC
PcWRKY11-OE-R	TCATTCCTTAGAAGATTGGAAAAC
PcWRKY11 - RNAi-F	CGTTTCTATCCTCCATCACAATCGG
PcWRKY11 - RNAi-R	GGGTGATGGTGCTCAGCCTCATAGG
62sk - proPcPIF3 - GUS-F	TAAAGTATGGGCACAATAAGATCG
62sk - proPcPIF3 - GUS-R	ACTACCAAATGTTAAAGCTTGAAGC
ABAi-proPcPIF3-200-F	gcttgaattcgagctcCATTATAAAAAGAAAAACTCACC
ABAi-proPcPIF3-200-R	cctcgaggtcgacTAGAATATCTCCAACAAACTCTATA
ABAi-proPcMYB10-800-F	gcttgaattcgagctcAGGCATCCACACCATCTGTAT
ABAi-proPcMYB10-800-R	cctcgaggtcgacGCATTCTGAATTTAACTCCACTT
ABAi-proPcUFGT-800-F	gcttgaattcgagctcGGTTTTCAGAATGCAGAATACCAGA

ABAi-proPcUFGT-800-R	cctcgaggtcgacCTCATTTAGCAGGAGCGAATCTTC
AD-PcWRKY40-F	ggccagtgaattcATGGACCATTCAGCTGCATAT
AD-PcWRKY40-R	gctcgatggatccTTAGTATGAATTGTGTTGAAGTATTCTTC
AD-PcWRKY53-F	ggccagtgaattcATGGAGAACTGCAATATACATTGGG
AD-PcWRKY53-R	gctcgatggatccTTAGGAAAAGAACCCTGGACCG
AD-PcPIF3-F	ggccagtgaattcATGCCTTTGTCCGAGCTTTAT
AD-PcPIF3-R	gctcgatggatccTTATCTGTTAGCTCTGTTGTTGTTA
AD-PcWRKY11-F	ggccagtgaattcATGGCTGTAGATCTAGTTGGCTTC
AD-PcWRKY11-R	gctcgatggatccTCATTCCTTAGAAGATTGGAAAAC
0800-proPcPIF3-F	tagaactagtggatccACACCTAATTCAGCAAACTTCGA
0800-proPcPIF3-R	cggtatcgataagcttTGTGAAGAGCTGGAAAATCACTG
0800-proPcUFGT-F	tagaactagtggatccGGTTTTCAGAATGCAGAATACCAGA
0800-proPcUFGT-R	cggtatcgataagcttTTGTTATACGGGAAAGTAGAAAGCA
0800-proPcMYB10-F	tagaactagtggatccAGAAGGCATCCACACCATCTGTAT
0800-proPcMYB10-R	cggtatcgataagcttCTAATTAATCTTCCAAGGCCAGTGA
2300-PcPIF3-F	agctcggtacccggggatccATGCCTTTGTCCGAGCTTTAT
2300-PcPIF3-R	CTTGCTCACCATGGTGTCGACTCTGTTAGCTCTGTTGTTGTTAGCA
2300-PcWRKY11-F	agctcggtacccggggatccATGGCTGTAGATCTAGTTGGCTTC
2300-PcWRKY11-R	CTTGCTCACCATGGTGTCGACTTCCTTAGAAGATTGGAAAAC

Supplemental Table S2. Gene bank of *WRKYs* and expression in the transcriptome which may bind to the W-BOX.

Gene	Description	FPKM_Starkrimson-75	FPKM_Red Bartlett-75	$\log_2$ (Red Bartlett-75/Starkrimson-75)
LOC103938975	probable WRKY transcription factor 40	194.463	65.8451	-1.56235
LOC103943771	probable WRKY transcription factor 53	51.8912	24.5566	-1.07938
LOC103945359	probable WRKY transcription factor 40	33.2326	7.10615	-2.22546
LOC103950478	probable WRKY transcription factor 11	35.3989	16.0009	-1.14555
LOC103957537	probable WRKY transcription factor 17	12.5875	5.07655	-1.31007

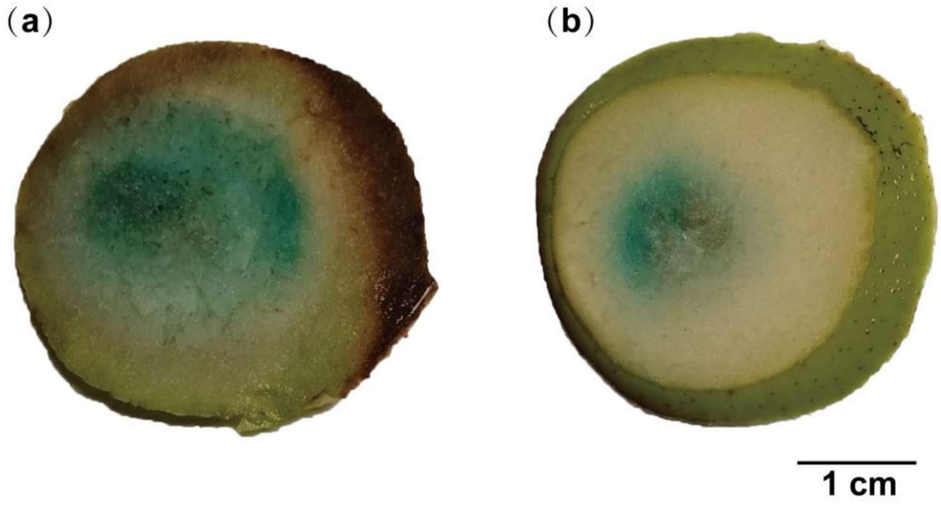

Supplemental Figure S1. GUS staining of the pear fruit.

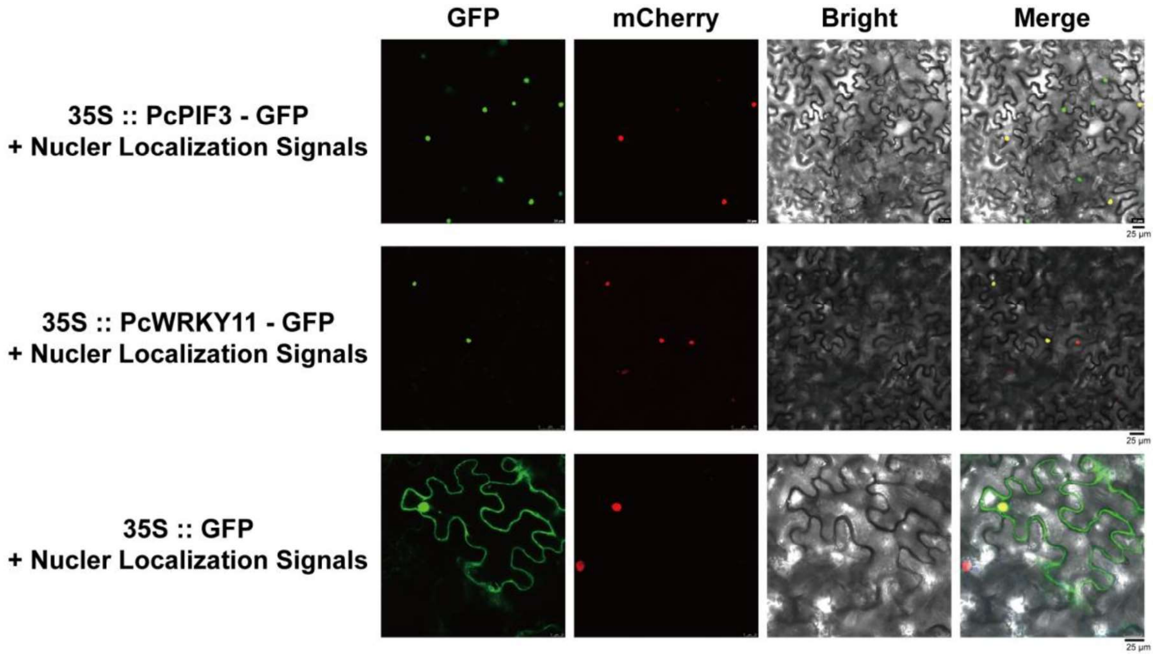

Supplemental Figure S2. Subcellular localization of PcPIF3 and PcWRKY11 expressed in tobacco leaf cells.

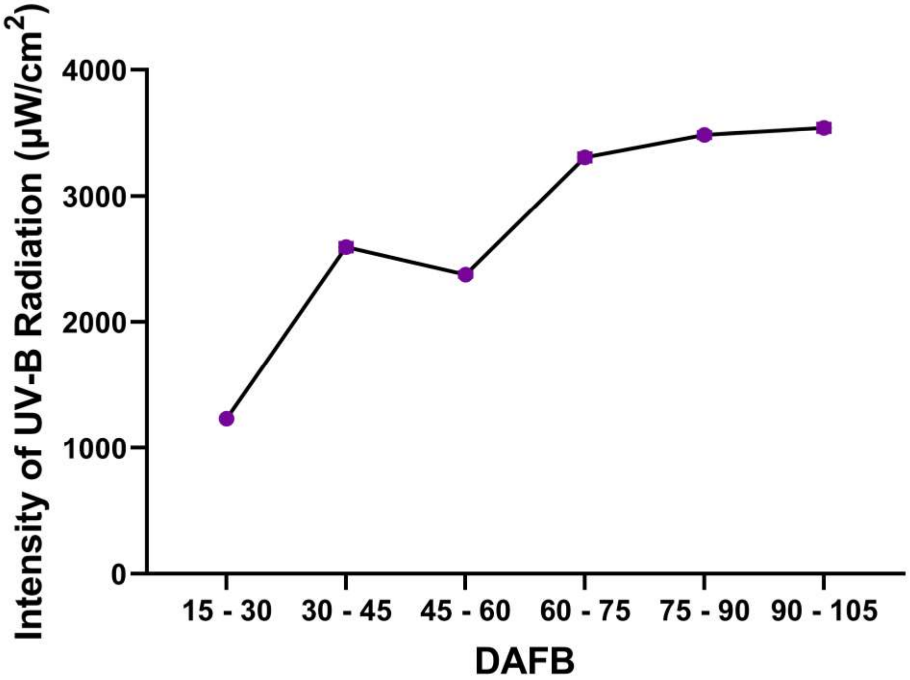

Supplemental Figure S3. Variation trend of UV-B radiation intensity with date.

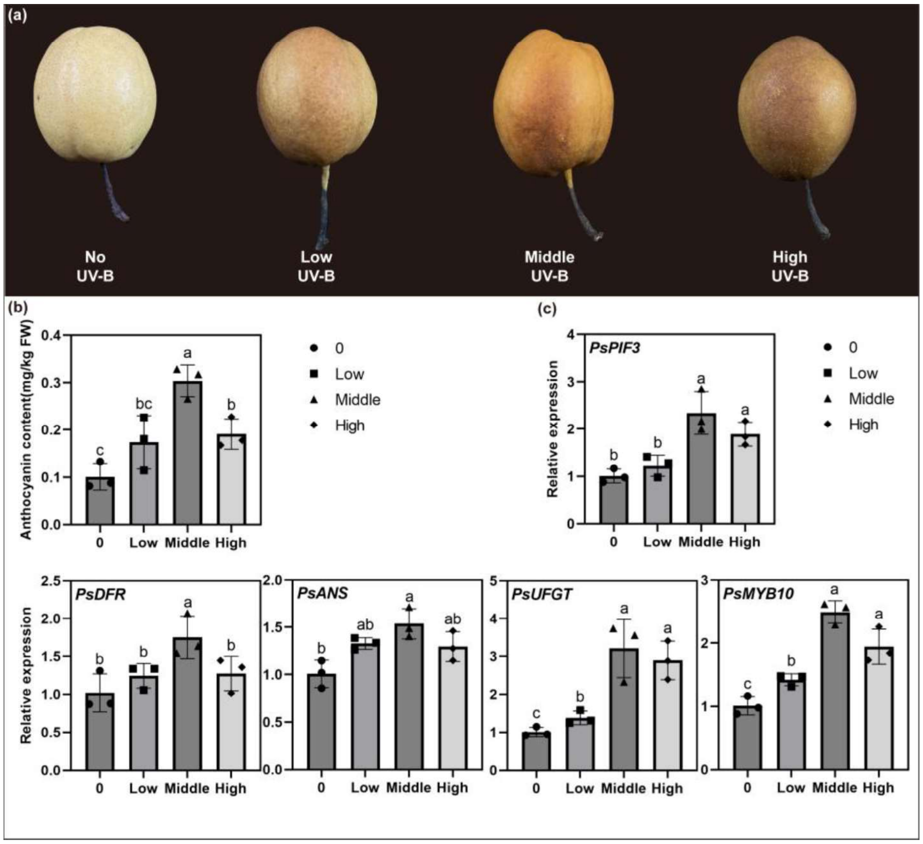

Supplemental Figure S4. Different intensities of UV-B irradiation were applied to pear fruits. (a) phenotypic changes; (b) anthocyanin content; (c) expression levels of related genes.

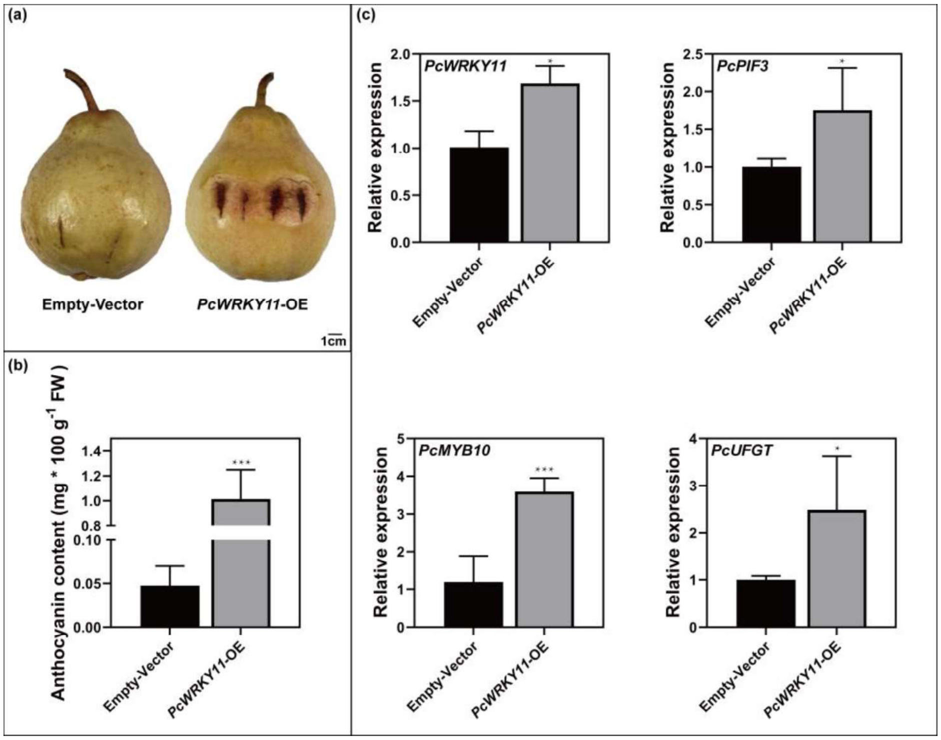

Supplemental Figure S5. Overexpression of *PcWRKY11* in 'Red Anjou' differences in anthocyanin and gene expression changes from controls.

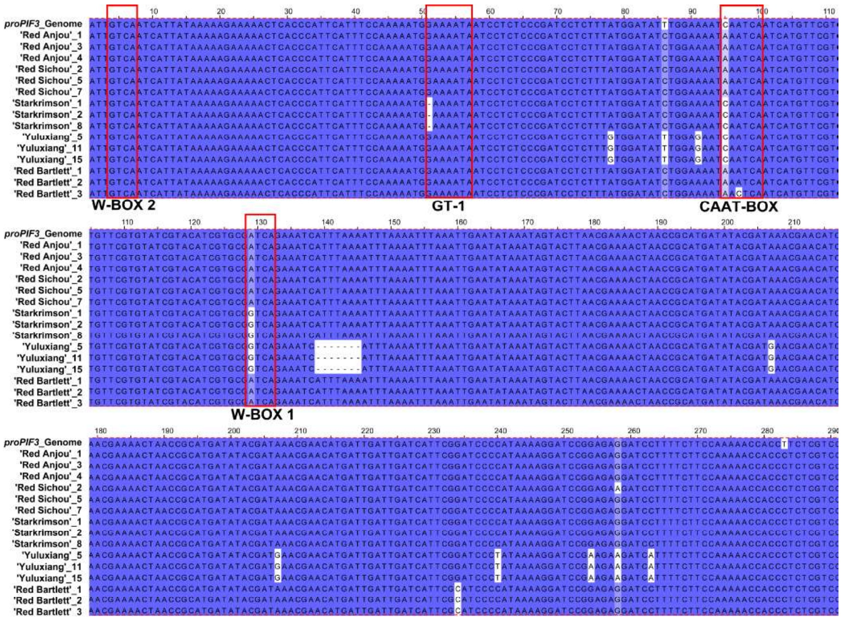

Supplemental Figure S6. The W-BOX element shared in the *PIF3* promoter region among pear varieties with different genetic backgrounds.

The authors have declared no conflict of interest.
